# Tissue iron is negatively correlated with TERC or TERT mRNA expression: a heterochronic parabiosis study in mice

**DOI:** 10.18632/aging.101676

**Published:** 2018-12-16

**Authors:** Meng-Wan Zhang, Peng Zhao, Wing-Ho Yung, Yuan Sheng, Ya Ke, Zhong-Ming Qian

**Affiliations:** 1 National Clinical Research Center for Aging and Medicine, Huashan Hostital, Laboratory of Neuropharmacology, School of Pharmacy, Fudan University, Shanghai 201203, PRC; 2 Laboratory of Neuropharmacology, Institute of Translational & Precision Medicine, Nantong University, Nantong 226019, PRC; 3 School of Biomedical Sciences and Gerald Choa Neuroscience Centre, Faculty of Medicine, The Chinese University of Hong Kong, Shatin, NT, Hong Kong

**Keywords:** heterochronic parabiosis, iron homeostasis, telomere and telomerase, liver, kidney and heart of mice, young and old mice

## Abstract

To test the hypothesis that iron accumulation in tissues with age is a key harmful factor for the development of aging, we established heterochronic parabiosis-pairings and investigated changes in serum iron, the expression of major iron transport proteins and iron contents, as well as telomerase reverse transcriptase (TERT), telomerase RNA component (TERC), and telomere length in the liver, kidney and heart of Y-O(O) (old pairing with young), Y-O(Y) (young pairing with old), O-O (pairings between two old) and Y-Y (pairings between two young) mice. We demonstrated that the reduced serum iron, increased iron and reduced expression of TERT and TERC in the tissues of aged mice are reversible by exposure to a younger mouse’s circulation. All of these measurements in young mice are reversible by exposure to an older mouse’s circulation. Correlation analysis showed that tissue iron is negatively correlated with TERT and TERC expression in the liver, kidney and heart of parabiotic mice. These findings provide new evidence for the key role of iron in aging and also imply the existence of rejuvenating factors in young serum with an anti-ageing role that act by reversing the impaired activity of iron metabolism in old mice.

## Introduction

Heterochronic parabiosis, which is the joining of the circulation systems of an aged mouse and a young mouse together, has been reported to have an anti-ageing effect. Studies of heterochronic parabiosis show that beneficial factors derived from the young systemic environment are able to activate molecular signaling pathways in hepatic, muscle or neural stem cells of the old parabiont, leading to increased tissue regeneration [1]. On the other hand, studies have also demonstrated that with age, the composition of the circulatory milieu changes in ways that broadly inhibit tissue regenerative capacities [2], suggesting the existence of certain harmful factors in the older organisms that trigger aging, thus preventing the rejuvenation process [2,3]. Currently, the beneficial or harmful factors involved in rejuvenation or aging and the relevant mechanisms are not completely understood, although chemokine CCL11 (eotaxin) [4], growth differentiation factor 11 (GDF11, a member of the TGF-β superfamily) [5–7], oxytocin [8], β-catenin [9], pro-inflammatory cytokines (most notably interleukin-6, IL-6) [10] and β2-microglobulin (B2M) [11] have been identified as some beneficial or harmful factors that are in part responsible for rejuvenating or aging effects.

Aging is characterized by a progressive loss of physiological integrity, leading to impaired function and increased vulnerability to death. This deterioration is the primary risk factor for major human pathologies, including cancer, diabetes, cardiovascular disorders, and neurodegenerative diseases [12]. The telomere is a validated biomarker of aging, comprising of multiple nucleotide repeats capping chromosomes [13]. The length of the telomere decreases with each cell division, eventually leading to cell senescence or apoptosis. Telomere shortening or damage is a driver of age-associated organ decline and disease risk [14]. Telomerase is a ribonucleoprotein enzyme complex that catalyzes the addition of telomeric repeats to chromosome ends, thereby counteracting the effects of telomere shortening [15]. This complex is essentially composed of telomerase RNA component (TERC) and telomerase reverse transcriptase (TERT). Deficiency in telomerase and telomeric proteins may lead to aging and senescence-associated disorders, while reactivation of endogenous telomerase activity can reverse tissue degeneration in aged telomerase-deficient mice [14].

Iron is an essential micronutrient which is required for many aspects of human physiology [16], including metabolic homeostasis and genome stability [17], while inflammation has a major impact on iron homeostasis. It has also been validated that iron is an extremely reactive transition metal that can interact with hydrogen peroxide to generate highly reactive and toxic hydroxyl radicals, thus stimulating oxidative stress and damage. Substantial evidence shows that oxidative stress and inflammation contribute to the attrition of the telomere and accelerate telomere shortening [18–20]. Indeed, iron-induced oxidative injury has been considered as a major factor for accelerated ageing, being associated with a number of age-related conditions and diseases [18,21,22]. Iron homeostasis and erythropoiesis regulate each other to ensure optimal delivery of oxygen and iron to cells and tissues [23], while hepcidin plays a central role in these two processes. The connection of most rejuvenating or aging factors identified, including CCL11 (eotaxin) [24], GDF11 [25,26], β-catenin [27,28], IL-6 [29–31] and B2M [32] with iron, erythropoiesis and hepcidin has been well documented.

The evidence discussed above led us to speculate that the accumulation of iron in tissues with age may be one of the key harmful factors in the development of aging. We also hypothesized that the beneficial factors in young animals may be able to reduce iron contents in tissues and then exhibit an anti-ageing effect. Therefore, here we established parabiotic pairings between young and old mice (heterochronic parabiosis), exposing old or young mice to factors present in young or old serum respectively, and investigated the changes in serum iron, the contents of iron, the expressions of major iron transport proteins as well as TERT and TERC, and telomere length in the liver, kidney and heart of Y-O(O) (old mice paired with young), Y-O(Y) (young mice paired with old), O-O (pairings between two old) and Y-Y (pairings between two young) mice. We demonstrated that the reduced iron in the serum, the increased iron contents and the reduced expression of TERT and TERC in tissues of aged mice are reversible by exposure to a younger mouse’s circulation. These measurements in young mice were also reversible by exposure to an older mouse’s circulation. Correlation analysis showed that tissue iron contents are negatively correlated with TERT and TERC expression in the liver, kidney and heart of heterochronic parabiotic mice. These findings provide key insights into understanding the key role of iron in aging.

## RESULTS

### Serum iron is significantly lower and tissue iron significantly higher in old compared to young mice

**Figure 1:**
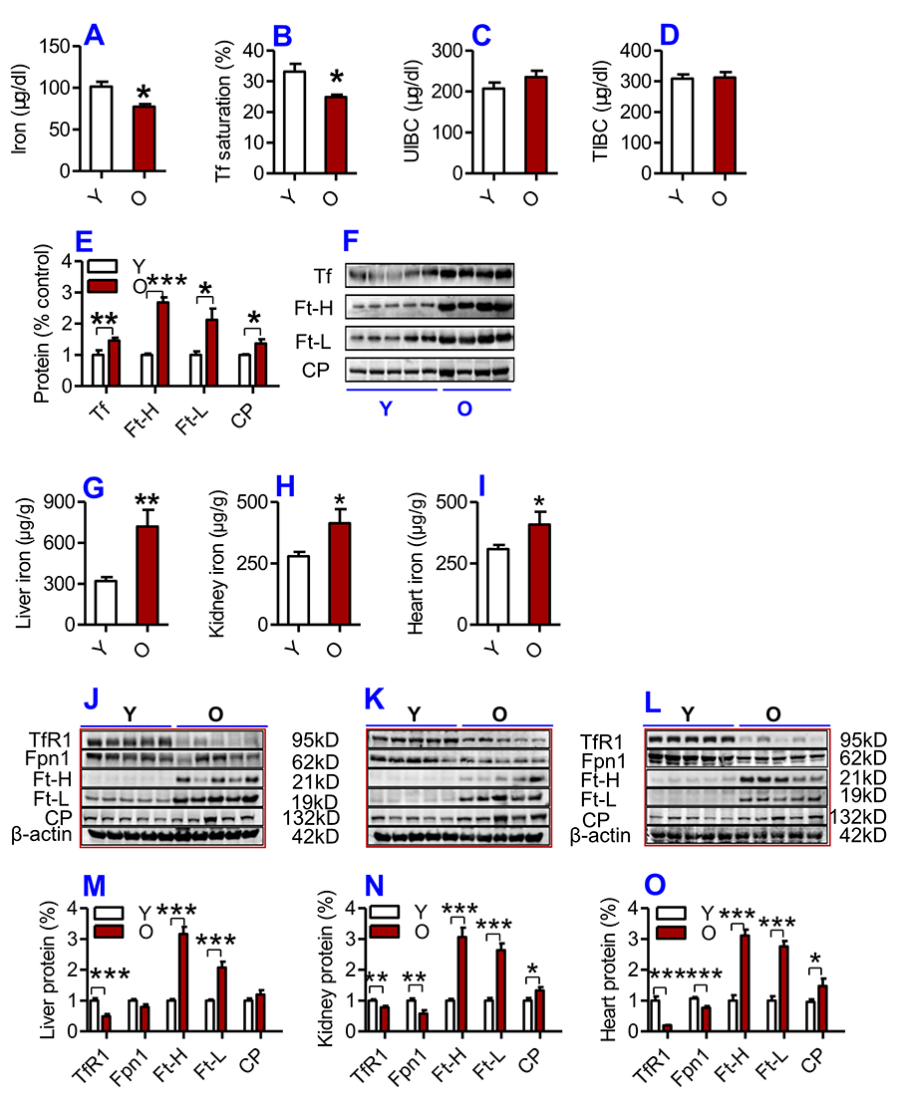
(**A-F**) Serum iron and other relevant indices in young and old mice. Serum iron (**A**), Tf saturation (**B**), UIBC (**C**), TIBC (**D**) and the contents of Tf, Ft-H, Ft-L, CP (**E** and **F**) were measured or calculated (Tf saturation and TIBC) in young (Y: 2-3 months) and old (O: 18-20 months) mice using commercial kits or western blot analysis as described in Methods and Materials. Data are presented as means ± SD (n=3). *p<0.05, **p<0.01 and ***p<0.001 vs. the control. (**G-L**) The contents of iron and the expression of iron metabolism proteins in the liver, kidney and heart of young and old mice. The contents of iron in the liver (**G**), kidney (**H**) and heart (**I**); the expression of TfR1, Fpn1, Ft-H, Ft-L and CP proteins in the liver (**J** and **M**), kidney (**K** and **N**) and heart (**L** and **O**) were determined in young (Y: 2-3 months) and old mice (O: 18-20 months) using western blot analysis or the methods described in Methods and Materials. Data are presented as means ± SEM (n=5). *p<0.05, **p<0.01 and ***p<0.001 vs. the control.

We first examined serum and tissue iron and other relevant indices in young (2-3 months) and old (18-20 months) C57BL/6 mice. Serum iron (Fig. 1A) and Tf saturation (Fig. 1B), but not UIBC (Fig. 1C) and TIBC (Fig. 1D), were significantly lower in old mice compared to young mice. The levels of both Ft-H and Ft-L in serum (biomarkers for body iron stores) were significantly higher in old mice than in young mice (Fig. 1E, F). Serum Tf (an iron carrier protein) and CP (a ferroxidase enzyme which was also connected to inflammation) were also significantly higher in old mice than in young mice (Fig. 1E, F).

The opposite was true for serum iron however; the contents of iron and the expression of both Ft-H and Ft-L in the liver (Fig. 1G; J & M), kidney (Fig. 1H; K & N) and heart (Fig. 1I, L &O) were found to be significantly higher in old mice compared to young mice. The levels of CP were significantly higher, while the expression of TfR1 (Cell-iron import protein) and Fpn1 (Cell-iron export protein) in the liver (Fig. 1J & M), kidney (Fig. 1K & N) and heart (Fig. 1L & O) were lower in old mice compared to young mice.

### Heterochronic parabiosis down-regulated serum iron level and up-regulated serum Ft-H, Ft-L, Tf and CP contents in young mice

**Figure 2:**
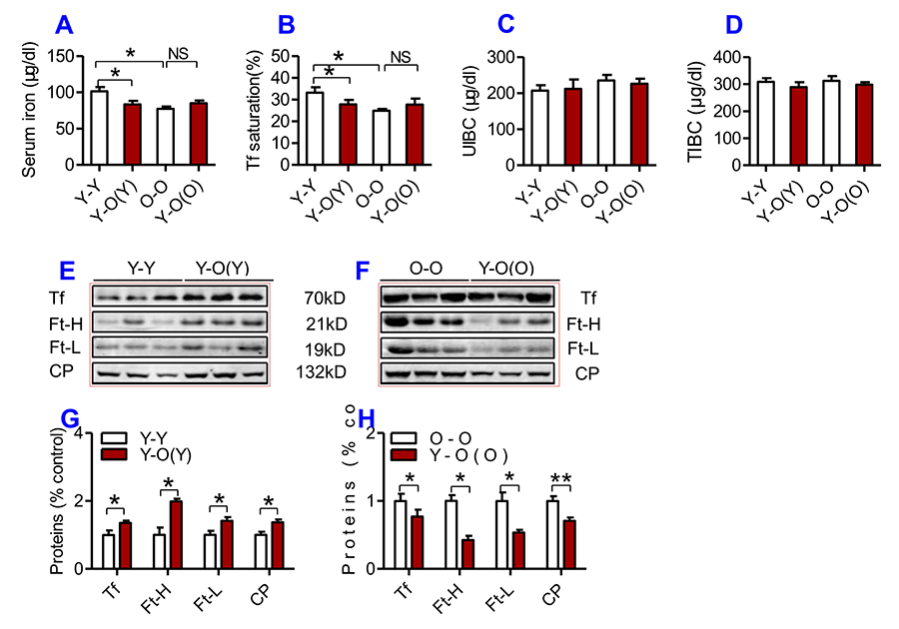
**The effects of heterochronic parabiosis on serum iron and other relevant indices in mice.** Serum iron (**A**), Tf saturation (**B**), UIBC (**C**), TIBC (**D**), and the contents of Tf, Ft-H, Ft-L, CP (**E-H**) were measured or calculated (Tf saturation and TIBC) in Y-Y (pairings between two young mice - isochronic parabiont), Y-O(Y) (young pairing with old – heterochronic parabiont), O-O (parings between two old - isochronic parabiont) and Y-O(O) (old pairing with young - heterochronic parabiont) mice using commercial kits or western blot analysis as described in Methods and Materials. Data are presented as means ± SEM (n=4). *p<0.05 and ** *p*<0.01 vs. the control.

**Figure 3:**
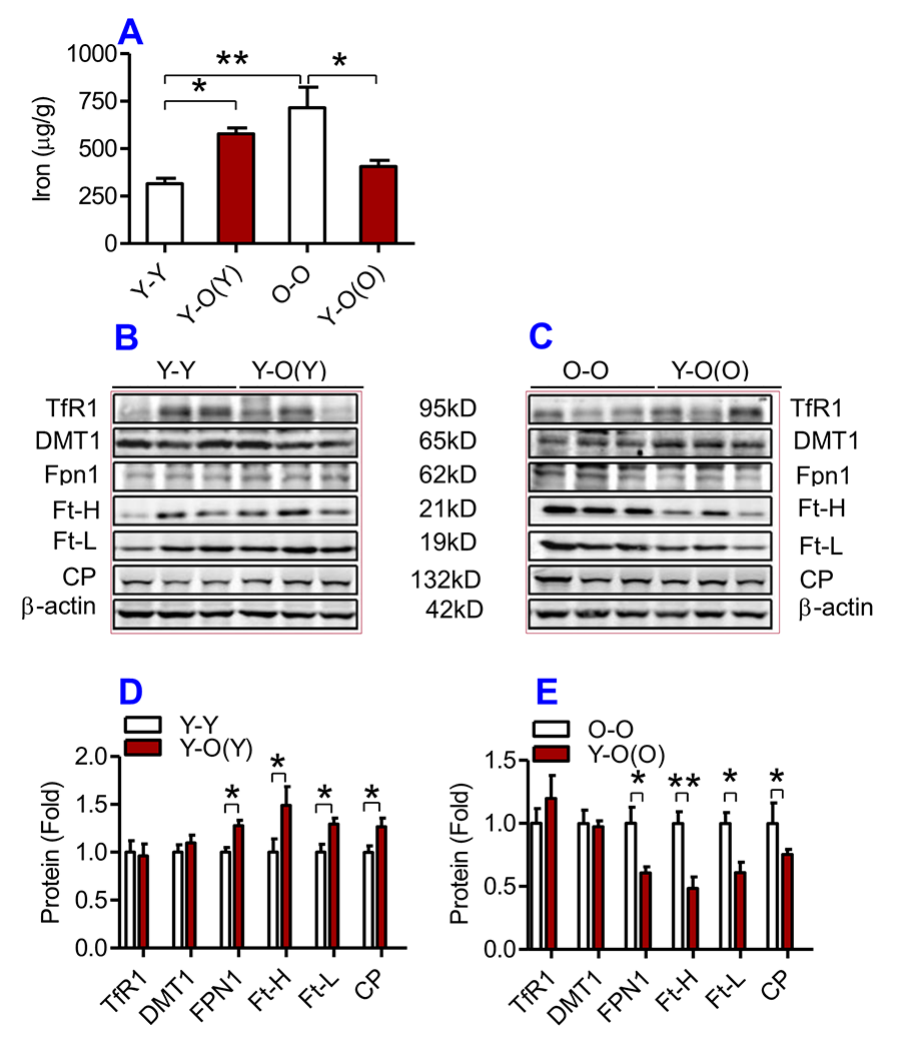
**The effects of heterochronic parabiosis on the contents of iron and the expression of iron metabolism proteins in the liver of mice.** The contents of iron (**A**), and the expression of TfR1, Fpn1, Ft-H, Ft-L and CP proteins (**B – E**) in the liver were determined in Y-Y, Y-O(Y), O-O and Y-O(O) mice using western blot analysis or the methods described previously. Data are presented as means ± SEM (n=3). *p<0.05 and **p<0.01 vs. the control.

To investigate the effects of heterochronic parabiosis on serum and tissue iron level, we connected a young mouse and an old mouse (Y-O) to build a heterochronic parabiotic model, and two young mice (Y-Y) or two old mice (O-O) to build an isochronic parabiotic model, for 4-weeks. Blood chimerism and the physiological effects of a heterochronic parabiotic model were then examined (Supplementary Figure 1). After 4 weeks, mice were euthanized and all designed measurements were conducted. After 4 weeks of exposure to the circulation of an old mouse, serum iron level (Fig. 2A) and Tf saturation (Fig. 2B) were significantly reduced, accompanied by increased serum Ft-H, Ft-L, Tf and CP contents (Fig. 2E & G) in young mice as compared to the isochronic parabiotic mice (Y-Y). Conversely, old mice parabiosed to young mice had increased serum iron level (Fig. 2A) and Tf saturation (Fig. 2B) although the differences were not significant, and reduced serum Ft-H, Ft-L, Tf and CP contents (Fig. 3F & H) as compared to the isochronic parabiotic mice (O-O). There were no significant differences in UIBC (Fig. 2C) and TIBC (Fig. 2D) among the Y-Y, Y-O(Y), O-O and Y-O(O) groups.

### Heterochronic parabiosis reduced the contents of iron, Ft and down-regulated the expression of Fpn1 and CP in the liver, kidney and heart in old mice

**Figure 4:**
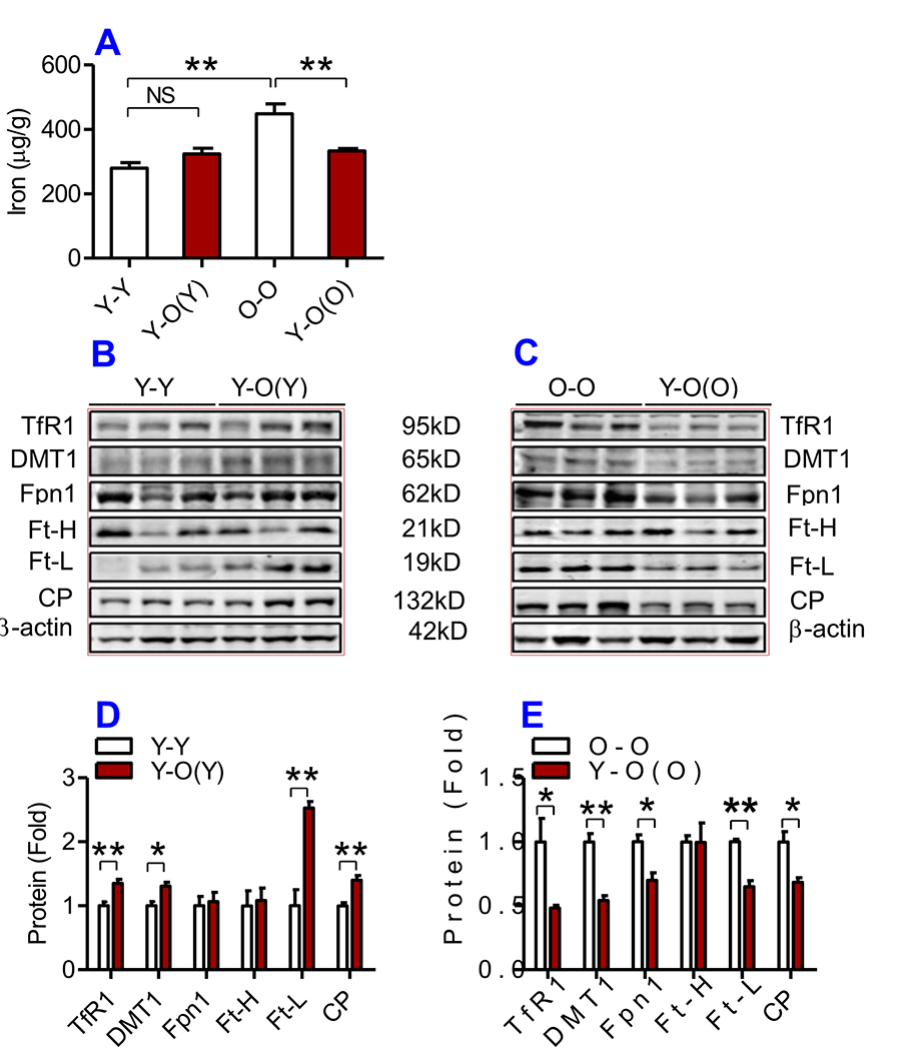
**The effects of heterochronic parabiosis on the contents of iron and the expression of iron metabolism proteins in the kidney of mice.** The contents of iron (**A**), and TfR1, Fpn1, Ft-H, Ft-L and CP proteins (**B – E**) in the kidney were determined in Y-Y, Y-O(Y), O-O and Y-O(O) mice using western blot analysis or the methods described previously. Data are presented as means ± SEM (n=4). *p<0.05 and **p<0.01 vs. the control.

**Figure 5:**
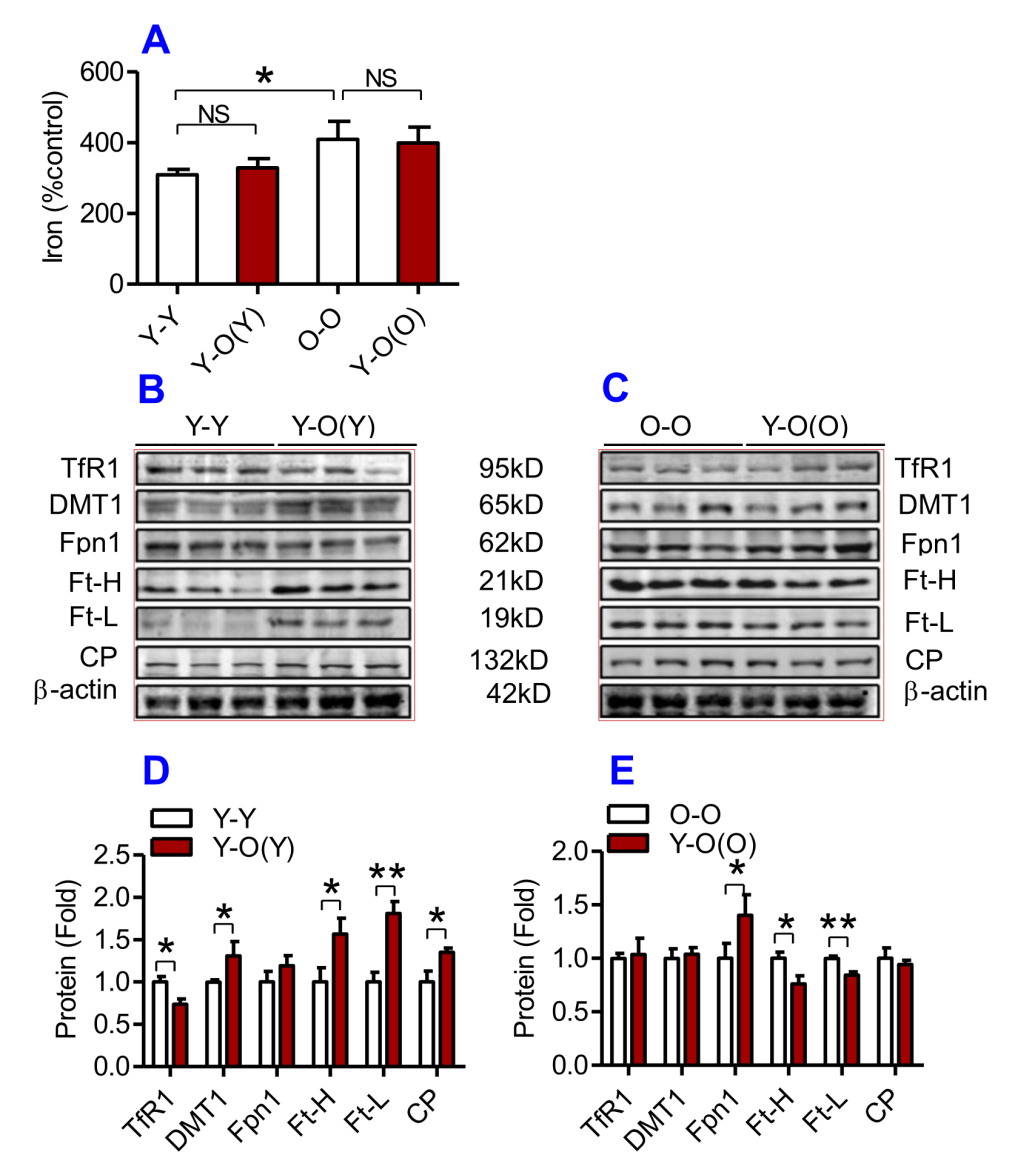
**The effects of heterochronic parabiosis on the contents of iron and the expression of iron metabolism proteins in the heart of mice.** The contents of iron (**A**), and the expression of TfR1, Fpn1, Ft-H, Ft-L and CP proteins (**B – E**) in the heart were determined in Y-Y, Y-O(Y), O-O and Y-O(O) mice using western blot analysis or the methods described previously. Data are presented as means ± SEM (n=4). *p<0.05 and **p<0.01 vs. the control.

We then examined the effects of heterochronic parabiosis on the contents of iron, Ft-H and Ft-L and the expression of TfR1, DMT1, Fpn1 and CP in the liver, kidney and heart. Old mice (Y-O(O)) parabiosed to young mice had reduced iron levels in the liver (Fig. 3A) and kidney (Fig. 4A), and reduced expression of Ft-H, Ft-L, Fpn1 and CP in the liver (Fig. 3C & E), reduced expression of Ft-L, TfR1, DMT1, Fpn1 and CP in the kidney (Fig. 4C & E), and reduced expression of Ft-H, Ft-L and Fpn1 in the heart (Fig. 5C & E), as compared with the isochronic parabiotic mice (O-O). There were no significant differences in iron content in the heart (Fig. 5A), the expression of TfR1 and DMT1 in the liver (Fig. 3C & E), Ft-H in the kidney (Fig. 4C & E), and TfR1, DMT1 and CP in the heart (Fig. 5C & E) between Y-O(O) and O-O mice.

In contrast, young mice parabiosed to old mice (Y-O(Y)) had increased iron level in the liver (Fig. 3A), increased Ft-H, Ft-L, Fpn1 and CP contents in the liver (Fig. 3B & D), increased Ft-L, TfR1, DMT1 and CP contents in the kidney (Fig. 4B & D), and increased Ft-H, Ft-L, DMT1, CP and decreased TfR1 in the heart (Fig. 5B & D), as compared with the isochronic parabiotic mice (Y-Y). Iron levels in the kidney (Fig. 4A), and heart (Fig. 5A) were also lower in Y-O(Y) mice than in Y-Y mice, although the differences were not significant. There were no significant differences in the expression of TfR1 and DMT1 in the liver (Fig. 3B & D), Ft-H and Fpn1 in the kidney (Fig. 4B & D), and Fpn1 in the heart (Fig. 5B & D) between Y-O(Y) and Y-Y mice.

### Heterochronic parabiosis down-regulated the expression of TERC and TERT mRNAs in young mice and up-regulated the expression of TERC and TERT mRNAs in old mice

**Figure 6:**
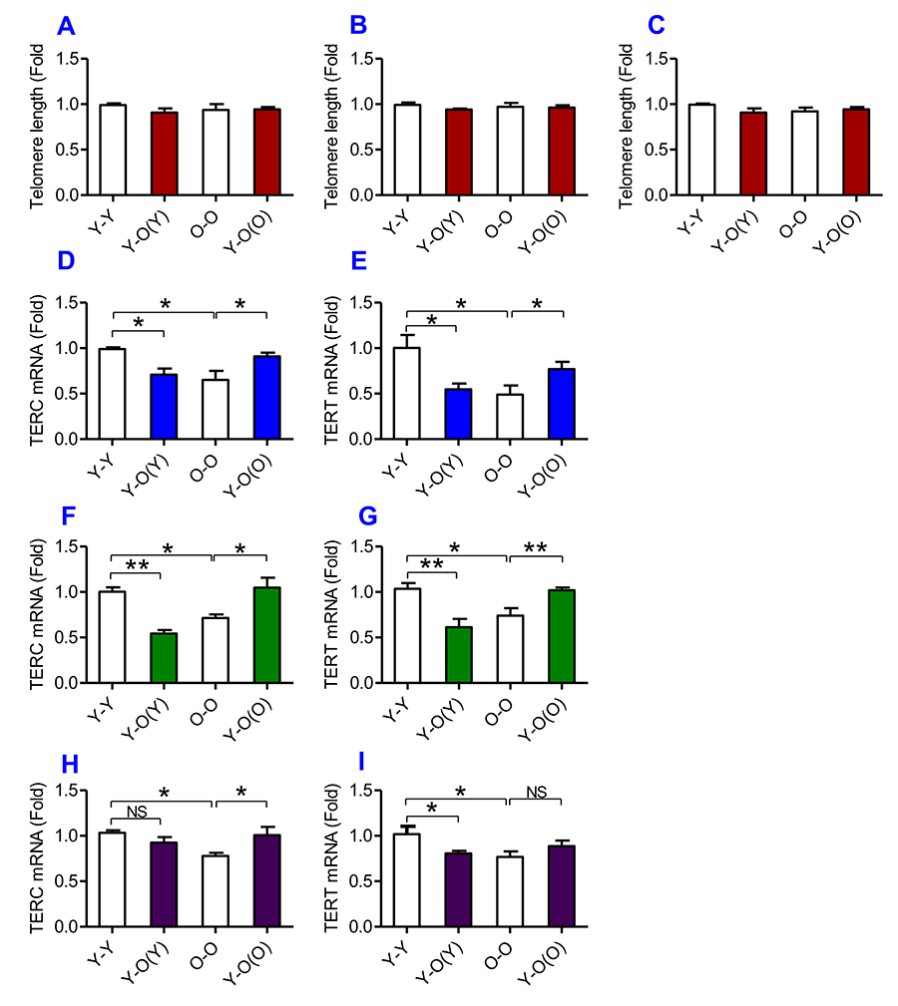
**The effects of heterochronic parabiosis on the length of the telomere and the expression of TERC or TERT mRNA in mice.** The telomere length in the liver (**A**), kidney (**B**) and heart (**C**) and the expression of TERC (**D, F** and **H**) or TERT mRNA (**E, G** and **I**) in the liver (**D** and **E**), kidney (**F** and **G**) and heart (**H** and **I**) were determined in Y-Y, Y-O(Y), O-O and Y-O(O) mice using real-time PCR as described in Methods and Materials. Data are presented as means ± SEM (n=3). *p<0.05 and **p<0.01 vs. the control.

To find out the effects of heterochronic parabiosis on telomere and telomerase, we measured telomere length and the expression of TERC and TERT in the liver, kidney and heart. We found that there were no significant differences in telomere length in the liver (Fig. 6A), kidney (Fig. 6B) and heart (Fig. 6C) among the Y-Y, Y-O(Y), O-O and Y-O(O) mice, suggesting that heterochronic parabiosis has no effect on telomere length. However, old mice parabiosed to young mice (Y-O(O)) had increased expression of TERC and TERT mRNAs in the liver (Fig. 6D & E), the kidney (Fig. 6F & G) and TERC mRNA in the heart (Fig. 6H) as compared with O-O mice. The expression of TERT mRNA in the heart (Fig. 6I) was also higher in Y-O(O) than in O-O mice, although the differences were not significant. Conversely, young mice parabiosed to old mice (Y-O(Y)) had reduced expression of TERC and TERT mRNAs in the liver (Fig. 6D & E) and kidney (Fig. 6F & G), and TERT mRNA in the heart (Fig. 6I), as compared with Y-Y mice. The expression of TERC mRNA in the heart (Fig. 6H) was also lower in Y-O(Y) than in Y-Y mice, although the difference was not significant.

### Tissue iron contents are negatively correlated with TERC or TERT mRNA expression

**Figure 7:**
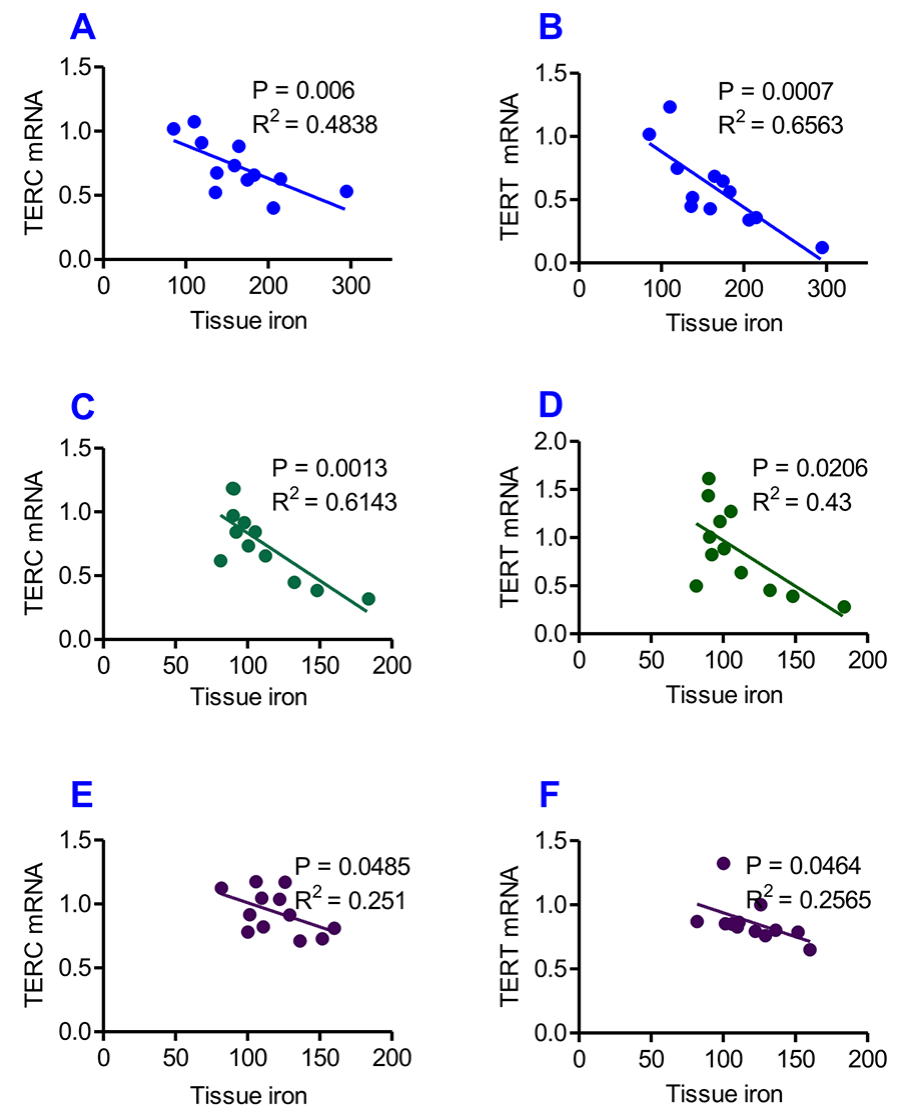
**Correlation analysis of the relationship between the contents of tissue iron and the expression of TERC or TERT mRNA in heterochronic parabiotic mice.** Correlation analysis of the content of iron and the expression of TERC or TERT mRNA in the liver (**A** and **B**), kidney (**C** and **D**) and heart (**E** and **F**) of heterochronic parabiotic mice was conducted by plotting the values for the relevant pairs against one another as described previously. Tissue iron contents were found to be negatively correlated with TERC or TERT mRNA expression in all-three organs examined.

Correlation analysis of the content of iron and the expression of TERC or TERT mRNA in the liver, kidney and heart of heterochronic parabiotic mice was conducted by plotting the values for the relevant pairs against one another as described previously [33,34]. Figure 7 lists the results of the correlation analysis. Highly significant correlations were found between the content of iron and the expression of TERC mRNA in the liver (Fig. 7A, R^2^ = 0.4838, P = 0.006), kidney (Fig. 7C, R^2^ = 0.6143, P = 0.0012) and heart (Fig. 7E, R^2^ = 0.2510, P = 0.0485), as well as between the content of iron and the expression TERT mRNA in the liver (Fig. 7B, R^2^ = 0.65638, P = 0.0007), kidney (Fig. 7D, R^2^ = 0.4300, P = 0.0206) and heart (Fig.7F, R^2^ = 0.2565, P = 0.0464).

## DISCUSSION

One of the major objectives of the present study was to find out whether exposure to a younger mouse’s serum is able to reduce iron contents in tissues and produce an anti-ageing effect in old mice. We demonstrated for the first time that heterochronic parabiosis (an old mouse sharing the same circulatory system with a young mouse) significantly reduced the contents of iron, Ft-H and Ft-L in the liver, kidney and heart, and up-regulated serum iron level in old mice. Also, heterochronic parabiosis up-regulated the expression of TERC and TERT mRNAs in the liver, kidney and heart in old mice. Furthermore, correlation analysis showed that tissue iron contents are negatively correlated with TERC or TERT mRNA expression in heterochronic parabiotic mice. These findings, plus the existing knowledge on the connection between iron and genome stability [17], iron-induced oxidative stress and telomere shortening or telomerase activity [18–20] and ageing [18] support the hypothesis that the accumulated iron in tissues with age is one of the key harmful factors in the development of aging, whereas reducing iron contents in tissues via exposure to a younger mouse’s serum produces an anti-ageing effect in old mice. Our results imply that there are beneficial factors in younger mice’s serum with an anti-ageing role that act by reversing impaired activity of iron metabolism in older mice.

On the other hand, we also found that heterochronic parabiosis could significantly increase the contents of iron, Ft-H and Ft-L in the liver, kidney and heart, and down-regulate serum iron level and up-regulate serum Ft-H and Ft-L contents in young mice. Also, heterochronic parabiosis down-regulated the expression of TERC and TERT mRNAs in the liver, kidney and heart of young mice. This data supports the notion that with age, the composition of the circulatory milieu changes in ways that broadly inhibit tissue regenerative capacity [2], and suggests the existence of certain harmful factors in the old serum which have a role in triggering aging and prevent the rejuvenation process by disrupting iron homeostasis.

Consistent with what was reported in a single heterochronic blood exchange study [35], we also found that in many cases, the inhibitory effects of old blood are more pronounced than the benefits of the young. Serum iron, Tf saturation, and the expression of TERC and TERT mRNAs in the kidney and heart in Y-O(Y) (young paired with old) mice all significantly differed from those in Y-Y (pairings between two young) mice, while these measurements in Y-O(O) (old paired with young) mice were not significantly different from those in O-O (pairing between two old) mice, although all of these indices were higher in Y-O(O) compared to O-O mice.

In most types of cells, iron balance is mainly dependent on the expression of four key cell-iron transporters, Fpn1 and CP (exporters), and TfR1 and DMT1 (importers). Fpn1 is a receptor for hepcidin, internalized and subsequently degraded after binding with hepcidin [36]. TfR1 has been found to be directly inhibited by hepcidin in different types of cells [37,38], and the ability of hepcidin to downregulate DMT1 expression has also been demonstrated in the intestine through proteasome internalization and degradation [39]. To clarify the possible mechanisms involved in the changes in tissue iron contents, we examined the effects of heterochronic parabiosis on the expression of TfR1, DMT1, Fpn1, and CP in the liver, kidney and heart in heterochronic parabiotic mice.

In the kidney, the expression of TfR1, DMT1 and CP was higher in Y-O(Y) than in Y-Y mice and the expression of TfR1, DMT1, Fpn1 and CP was lower in Y-O(O) than in O-O mice. Increased expression in TfR1 and DMT1 would lead to an increase in iron uptake by cells, which might be one of the reasons for the increased iron content in the kidney of Y-O(Y) mice as compared with Y-Y mice. In Y-O(O) mice, the expression of both importers (TfR1 and DMT1) and exporters (Fpn1 and CP) was inhibited, which would induce a reduction in iron uptake as well as release in the cells [36]. One possible explanation for why iron contents were lower in Y-O(O) compared to O-O mice is that the inhibitory effect on TfR1 and DMT1 expression might be more pronounced than that on Fpn1 and CP. In the heart, the increased expression of TfR1 and DMT1 was likely to be partly associated with increased iron contents in Y-O(Y) mice when compared with Y-Y mice, while the reduced iron content in Y-O(O) mice compared to O-O mice may be due to increased iron release induced by the increased expression of Fpn1. However, changes in the expression of iron transporters in the liver cannot explain the effects of heterochronic parabiosis on tissue iron contents. No difference was found in the expressions of TfR1 and DMT1 between Y-O(Y) and Y-Y mice, as well as between Y-O(O) and O-O mice, while the expression of Fpn1 and CP was higher in Y-O(Y) than in Y-Y mice and lower in Y-O(O) than in O-O mice. The increased expression of Fpn1 in Y-O(Y) mice should have induced an increase in iron release and a reduction in cell iron [36], iron contents however were higher rather than lower in Y-O(Y) compared to Y-Y mice. The same was also found in Y-O(O) and O-O mice; further investigation on this issue is needed.

A number of studies have reported that telomeres in primary human cells shorten with age, both in vitro and in vivo [40–43]. It has also been confirmed that the non-coding sequences at the ends of chromosomes progressively shorten with each cell division in the absence of telomerase [43]. In present study, we found that young mice parabiosed to old mice had reduced expression of TERC and TERT mRNAs in the liver and kidney, and TERT mRNA in the heart. The reduction in telomerase expression should theoretically result in a reduction in telomere length, but our data showed that there were no significant differences in telomere length in the liver, kidney and heart among the Y-Y, Y-O(Y), O-O and Y-O(O) mice. The underlying reason is unknown, however it is possible that the changes in the activity of telomerase induced by heterochronic parabiosis did not reach a level that could induce a visibly significant change in telomere length under our experimental conditions

It will be important to identify the key factors responsible for these beneficial or harmful effects on iron metabolism in mice serum, as identification of these factors would make a critical contribution to better understanding the precise role of iron in ageing and the relevant mechanisms involved in the iron-mediated ageing process. Mammalian iron metabolism is regulated systemically by hepcidin [44]. Therefore, it is absolutely needed to clarify the effects of heterochronic parabiosis on the expression of this iron regulation hormone. The changes in the expression of CCL11 (eotaxin), GDF11, β-catenin, IL-6 and B2M under our experimental conditions are also worthy to be investigated, because these reported “beneficial or harmful factors” have been connected with iron, erythropoiesis and hepcidin iron metabolism [24–32]. In addition, although Baytril was used to control infection in the present study, it is important to evaluate the effects of parabiosis on markers of inflammation and on the cellular localization of iron under our experimental conditions, because inflammation can be an important factor in the changes in iron levels. These and other relevant studies are currently under the way in our laboratories.

In conclusion, in the present study we demonstrate for the first time that iron contents in the liver, kidney and heart are negatively correlated with TERC or TERT mRNA expression in heterochronic parabiotic mice, providing further evidence to support the notion that the accumulated iron in tissues with age is one of the key harmful factors in the ageing process. Our results also imply the existence of some beneficial or rejuvenating factors in serum of younger mice with an anti-ageing role that act by reversing the impaired activity of iron metabolism in old mice, and also the existence of certain harmful factors in the old serum which have a role in triggering aging and prevent the rejuvenation process by disrupting iron homeostasis in young mice.

## MATERIALS AND METHODS

### Materials

Unless otherwise stated, all chemicals were obtained from the Sigma Chemical Company, St. Louis, MO, USA. Mouse monoclonal anti-rat transferrin receptor 1 (TfR1) was purchased from Invitrogen, Carlsbad, CA, USA; rabbit polyclonal anti-rat divalent metal transporter 1 (DMT1, SLC11A2) and rabbit polyclonal anti ferritin-light-chain (Ft-L) from Proteintech, Chicago, IL, USA; rabbit polyclonal anti ferritin-heavy-chain (Ft-H) from Bioworld Technology Inc., Louis Park, MN, USA; rabbit polyclonal anti-mouse ferroportin 1 (Fpn1) from Novus Biologicals, Littleton, CO, USA; mouse anti-transferrin (Tf) and anti-ceruloplasmin (CP) from Alpha Diagnostic International Company, San Antonio, TX, USA. Goat anti-rabbit or anti-mouse IRDye 800 CW secondary antibody was purchased from Li-Cor, Lincoln, NE, USA; TRIzol reagent from Life Technologies, Carlsbad, CA, USA; and RevertAid First Strand cDNA Synthesis Kit and BCA Protein Assay Kit both from Thermo Scientific, Waltham, MA, USA.

### Animals

C57BL/6 mice were bred and maintained at the Animal Holding Unit of Fudan University School of Pharmacy. CAG-GFP (CAG-green fluorescent protein) transgenic mice were purchased from the Shanghai Model Organisms Center, Shanghai, China. All animal care and experimental protocols were performed according to the Animal Management Rules of the Ministry of Health of China, and approved by the Animal Ethics Committees of Fudan University and The Chinese University of Hong Kong.

### Parabiosis and flow cytometry

Female mice, aged 2-3 months (young) and 18-20 months (old), were randomly assigned to establish parabiotic pairings between young and old (Y-O) mice (heterochronic parabiosis, HP), with parabiotic pairings between two young mice (Y-Y) or two old (O-O) mice (isochronic parabiosis, IP) as controls. The GFP+ mice were used in the present study, and not only in the pilot studies for developing the parabiosis model (Supplementary Figure 1). Parabiosis surgery was conducted according to Conboy et al. [45]. In parabiosis, animals develop vascular anastomoses and thus a single, shared circulatory system [41]. Mirror-image incisions at the left and right flanks were made through the skin respectively. Shorter incisions were made through the abdominal wall. The peritoneal openings of the adjacent parabionts were sutured together. Elbow and knee joints from each parabiont were sutured together and the skin of each mouse was stapled to the skin of the adjacent parabiont. Each mouse was injected subcutaneously with Baytril (a broad-spectrum antibiotic) and Buprenex as directed for pain, and monitored during recovery. Flow cytometric analysis was done on fixed and permeabilized blood plasma cells from GFP and non-GFP parabionts according to Villeda et al. [4]. The changes in body weight and the viabilities of the pairs were also observed. The survival rate for parabionts was 53% in the present study.

### Sampling of blood and tissues

Animals were anesthetized with 1% pentobarbital sodium (40 mg/kg body weight, intraperitoneally) and decapitated. Blood samples were collected into heparinized syringes for the determination of serum iron (SI), unsaturated iron-binding capacity (UIBC), total iron-binding capacity (TIBC), and transferrin saturation (TS). Mice were then perfused with phosphate-buffered saline (PBS), the liver, heart, and kidney were removed, excised, and rinsed in PBS, before being dried and weighed [46,47] for total RNA extraction, protein determination, and iron measurement.

### Serum iron and transferrin saturation

SI and UIBC were measured using commercial kits as described [48,49]. TIBC (micrograms per deciliter TIBC = SI + UIBC) and transferrin saturation (TS = SI/TIBC x 100) were calculated.

### Tissue iron measurement

Tissues were dried and weighed for iron measurement. Tissue iron contents (μg/g wet weight of tissue) in the liver, kidney or heart were measured according to methods described previously [50].

### Western blot analysis

The tissue was washed and homogenized were prepared as described [51]. Aliquots of the extract containing about 30 μg of protein were loaded and run on a single track of 12% (for Ft-H and Ft-L), 8% (for CP), or 10% (for others) sodium dodecyl sulfate-polyacrylamide gel electrophoresis under reducing conditions before being subsequently transferred to a pure nitrocellulose membrane. The blots were blocked and then incubated with primary antibodies: mouse anti-Tf (1:500), anti-CP (1:500), anti-TfR1 (1:500), rabbit anti-Fpn1 (1:1000), rabbit anti-DMT1 (1:1000), rabbit anti-Ft-L (1:1000), and rabbit anti-Ft-H (1:1000), overnight at 4°C. After being washed, the blots were incubated with goat anti-rabbit or anti-mouse IRDye 800 CW secondary antibody (1:20,000) for 2 hours at 37°C. The intensity of the specific bands was detected and analyzed with the Odyssey infrared imaging system (Li-Cor). To ensure even loading of the samples, the same membrane was probed with rabbit anti-β-actin polyclonal antibody at a 1:2000 dilution [52]. The contents of Tf, Ft-H, Ft-L, CP were also measured by the above procedure and Ponceau staining was used for protein loading control as described by Rivero-Gutiérrez et al [53].

### Isolation of total RNA and quantitative real-time PCR

Total RNA extraction and complementary DNA preparation were respectively performed using TRIzol reagent and the RevertAid First Strand cDNA Synthesis Kit in accordance with the instructions of the manufacturers. Real-time PCR was carried out using FastStart Universal SYBR Green Master and Light-Cycler96. The specific pairs of primers of mouse β-actin, telomerase RNA component (TERC), and telomerase reverse transcriptase (TERT) are listed in Supplementary Table 1. The cycle threshold value of each target gene was normalized to that of the β-actin mRNA. Relative gene expression was calculated by the 2^–ΔΔCT^ method [54,55].

### Telomere length measurement by real-time PCR

Genomic DNA was extracted from liver, kidney and heart tissue using an E.Z.N.A Tissue DNA kit (Omega, US). DNA concentration was adjusted to 5 ng/l in H_2_O. Telomere PCR reaction conditions were 3 minutes at 95°C followed by 40 cycles of 15 seconds at 95°C and 1 minute at 54°C, with 300 nm Telomere primers (Supplementary Table 1). Real-time PCR was carried out using FastStart Universal SYBR Green Master and LightCycler96 with 10 ng genomic DNA. The PCR assay calculates the ratio between telomeric repeat copy number (T) and that of a single reference gene (36B4) (S). Relative T/S is calculated in relation to a reference curve and final measurements are exponentiated to ensure normality) [56,57]. All telomeric and 36B4 reactions were measured in triplicate, and the average was used for final calculations.

### Statistical analysis

Statistical analysis was performed using one-way analysis of variance (ANOVA) and Tukey method was used for multiple pair-wise comparisons. All data are expressed as the mean ± SEM. Values of *p* < 0.05 were taken to be statistically significant.

## SUPPLEMENTARY MATERIAL

Supplementary Figure 1

Supplementary Table 1

## References

[1] Conese M, Carbone A, Beccia E, Angiolillo A. The fountain of youth: a tale of parabiosis, stem cells, and rejuvenation. Open Med (Wars). 2017; 12:376–83. 10.1515/med-2017-0053 29104943 PMC5662775

[2] Liu Y, Conboy MJ, Mehdipour M, Liu Y, Tran TP, Blotnick A, Rajan P, Santos TC, Conboy IM. Application of bio-orthogonal proteome labeling to cell transplantation and heterochronic parabiosis. Nat Commun. 2017; 8:643. 10.1038/s41467-017-00698-y 28935952 PMC5608760

[3] Pishel I, Shytikov D, Orlova T, Peregudov A, Artyuhov I, Butenko G. Accelerated aging versus rejuvenation of the immune system in heterochronic parabiosis. Rejuvenation Res. 2012; 15:239–48. 10.1089/rej.2012.1331 22533440

[4] Villeda SA, Luo J, Mosher KI, Zou B, Britschgi M, Bieri G, Stan TM, Fainberg N, Ding Z, Eggel A, Lucin KM, Czirr E, Park JS, et al. The ageing systemic milieu negatively regulates neurogenesis and cognitive function. Nature. 2011; 477:90–94. 10.1038/nature10357 21886162 PMC3170097

[5] Loffredo FS, Steinhauser ML, Jay SM, Gannon J, Pancoast JR, Yalamanchi P, Sinha M, Dall’Osso C, Khong D, Shadrach JL, Miller CM, Singer BS, Stewart A, et al. Growth differentiation factor 11 is a circulating factor that reverses age-related cardiac hypertrophy. Cell. 2013; 153:828–39. 10.1016/j.cell.2013.04.015 23663781 PMC3677132

[6] Sinha M, Jang YC, Oh J, Khong D, Wu EY, Manohar R, Miller C, Regalado SG, Loffredo FS, Pancoast JR, Hirshman MF, Lebowitz J, Shadrach JL, et al. Restoring systemic GDF11 levels reverses age-related dysfunction in mouse skeletal muscle. Science. 2014; 344:649–52. 10.1126/science.1251152 24797481 PMC4104429

[7] Chen S, Feng T, Vujić Spasić M, Altamura S, Breitkopf-Heinlein K, Altenöder J, Weiss TS, Dooley S, Muckenthaler MU. Transforming Growth Factor β1 (TGF-β1) activates hepcidin mRNA expression in hepatocytes. J Biol Chem. 2016; 291:13160–74. 10.1074/jbc.M115.691543 27129231 PMC4933231

[8] Elabd C, Cousin W, Upadhyayula P, Chen RY, Chooljian MS, Li J, Kung S, Jiang KP, Conboy IM. Oxytocin is an age-specific circulating hormone that is necessary for muscle maintenance and regeneration. Nat Commun. 2014; 5:4082. 10.1038/ncomms5082 24915299 PMC4512838

[9] Baht GS, Silkstone D, Vi L, Nadesan P, Amani Y, Whetstone H, Wei Q, Alman BA. Exposure to a youthful circulaton rejuvenates bone repair through modulation of β-catenin. Nat Commun. 2015; 6:7131. 10.1038/ncomms8131 25988592 PMC4479006

[10] Eisenstaedt R, Penninx BW, Woodman RC. Anemia in the elderly: current understanding and emerging concepts. Blood Rev. 2006; 20:213–26. 10.1016/j.blre.2005.12.002 16472893

[11] Smith LK, He Y, Park JS, Bieri G, Snethlage CE, Lin K, Gontier G, Wabl R, Plambeck KE, Udeochu J, Wheatley EG, Bouchard J, Eggel A, et al. β2-microglobulin is a systemic pro-aging factor that impairs cognitive function and neurogenesis. Nat Med. 2015; 21:932–37. 10.1038/nm.3898 26147761 PMC4529371

[12] López-Otín C, Blasco MA, Partridge L, Serrano M, Kroemer G. The hallmarks of aging. Cell. 2013; 153:1194–217. 10.1016/j.cell.2013.05.039 23746838 PMC3836174

[13] Verma S, Tachtatzis P, Penrhyn-Lowe S, Scarpini C, Jurk D, Von Zglinicki T, Coleman N, Alexander GJ. Sustained telomere length in hepatocytes and cholangiocytes with increasing age in normal liver. Hepatology. 2012; 56:1510–20. 10.1002/hep.25787 22504828

[14] Jaskelioff M, Muller FL, Paik JH, Thomas E, Jiang S, Adams AC, Sahin E, Kost-Alimova M, Protopopov A, Cadiñanos J, Horner JW, Maratos-Flier E, Depinho RA. Telomerase reactivation reverses tissue degeneration in aged telomerase-deficient mice. Nature. 2011; 469:102–06. 10.1038/nature09603 21113150 PMC3057569

[15] Brown KE, Meleah Mathahs M, Broadhurst KA, Coleman MC, Ridnour LA, Schmidt WN, Spitz DR. Increased hepatic telomerase activity in a rat model of iron overload: a role for altered thiol redox state? Free Radic Biol Med. 2007; 42:228–35. 10.1016/j.freeradbiomed.2006.10.039 17189828 PMC1820590

[16] Ke Y, Qian ZM. Brain iron metabolism: neurobiology and neurochemistry. Prog Neurobiol. 2007; 83:149–73. 10.1016/j.pneurobio.2007.07.009 17870230

[17] Prá D, Franke SI, Henriques JA, Fenech M. Iron and genome stability: an update. Mutat Res. 2012; 733:92–99. 10.1016/j.mrfmmm.2012.02.001 22349350

[18] Polla AS, Polla LL, Polla BS. Iron as the malignant spirit in successful ageing. Ageing Res Rev. 2003; 2:25–37. 10.1016/S1568-1637(02)00048-X 12437994

[19] von Zglinicki T, Martin-Ruiz CM. Telomeres as biomarkers for ageing and age-related diseases. Curr Mol Med. 2005; 5:197–203. 10.2174/1566524053586545 15974873

[20] Houben JM, Moonen HJ, van Schooten FJ, Hageman GJ. Telomere length assessment: biomarker of chronic oxidative stress? Free Radic Biol Med. 2008; 44:235–46. 10.1016/j.freeradbiomed.2007.10.001 18021748

[21] Qian ZM, Shen X. Brain iron transport and neurodegeneration. Trends Mol Med. 2001; 7:103–08. 10.1016/S1471-4914(00)01910-9 11286780

[22] Ke Y, Ming Qian Z. Iron misregulation in the brain: a primary cause of neurodegenerative disorders. Lancet Neurol. 2003; 2:246–53. 10.1016/S1474-4422(03)00353-3 12849213

[23] Kim A, Nemeth E. New insights into iron regulation and erythropoiesis. Curr Opin Hematol. 2015; 22:199–205. 10.1097/MOH.0000000000000132 25710710 PMC4509743

[24] Bournazou I, Mackenzie KJ, Duffin R, Rossi AG, Gregory CD. Inhibition of eosinophil migration by lactoferrin. Immunol Cell Biol. 2010; 88:220–23. 10.1038/icb.2009.86 19918259

[25] Dussiot M, Maciel TT, Fricot A, Chartier C, Negre O, Veiga J, Grapton D, Paubelle E, Payen E, Beuzard Y, Leboulch P, Ribeil JA, Arlet JB, et al. An activin receptor IIA ligand trap corrects ineffective erythropoiesis in β-thalassemia. Nat Med. 2014; 20:398–407. 10.1038/nm.3468 24658077 PMC7730561

[26] Suragani RN, Cadena SM, Cawley SM, Sako D, Mitchell D, Li R, Davies MV, Alexander MJ, Devine M, Loveday KS, Underwood KW, Grinberg AV, Quisel JD, et al. Transforming growth factor-β superfamily ligand trap ACE-536 corrects anemia by promoting late-stage erythropoiesis. Nat Med. 2014; 20:408–14. 10.1038/nm.3512 24658078

[27] Chiang CL, Chen SS, Lee SJ, Tsao KC, Chu PL, Wen CH, Hwang SM, Yao CL, Lee H. Lysophosphatidic acid induces erythropoiesis through activating lysophosphatidic acid receptor 3. Stem Cells. 2011; 29:1763–73. 10.1002/stem.733 21915944

[28] Song S, Christova T, Perusini S, Alizadeh S, Bao RY, Miller BW, Hurren R, Jitkova Y, Gronda M, Isaac M, Joseph B, Subramaniam R, Aman A, et al. Wnt inhibitor screen reveals iron dependence of β-catenin signaling in cancers. Cancer Res. 2011; 71:7628–39. 10.1158/0008-5472.CAN-11-2745 22009536

[29] Nemeth E, Rivera S, Gabayan V, Keller C, Taudorf S, Pedersen BK, Ganz T. IL-6 mediates hypoferremia of inflammation by inducing the synthesis of the iron regulatory hormone hepcidin. J Clin Invest. 2004; 113:1271–76. 10.1172/JCI200420945 15124018 PMC398432

[30] Qian ZM, He X, Liang T, Wu KC, Yan YC, Lu LN, Yang G, Luo QQ, Yung WH, Ke Y. Lipopolysaccharides upregulate hepcidin in neuron via microglia and the IL-6/STAT3 signaling pathway. Mol Neurobiol. 2014; 50:811–20. 10.1007/s12035-014-8671-3 24659348

[31] Zhang FL, Hou HM, Yin ZN, Chang L, Li FM, Chen YJ, Ke Y, Qian ZM. Impairment of Hepcidin upregulation by lipopolysaccharide in the Interleukin-6 knockout mouse brain. Front Mol Neurosci. 2017; 10:367. 10.3389/fnmol.2017.00367 29163042 PMC5681933

[32] Muckenthaler M, Roy CN, Custodio AO, Miñana B, deGraaf J, Montross LK, Andrews NC, Hentze MW. Regulatory defects in liver and intestine implicate abnormal hepcidin and Cybrd1 expression in mouse hemochromatosis. Nat Genet. 2003; 34:102–07. 10.1038/ng1152 12704390

[33] Li Z, Lai Z, Ya K, Fang D, Ho YW, Lei Y, Ming QZ. Correlation between the expression of divalent metal transporter 1 and the content of hypoxia-inducible factor-1 in hypoxic HepG2 cells. J Cell Mol Med. 2008; 12:569–79. 10.1111/j.1582-4934.2007.00145.x 18419598 PMC3822544

[34] Yang G, Hu R, Zhang C, Qian C, Luo QQ, Yung WH, Ke Y, Feng H, Qian ZM. A combination of serum iron, ferritin and transferrin predicts outcome in patients with intracerebral hemorrhage. Sci Rep. 2016; 6:21970. 10.1038/srep21970 26898550 PMC4761997

[35] Rebo J, Mehdipour M, Gathwala R, Causey K, Liu Y, Conboy MJ, Conboy IM. A single heterochronic blood exchange reveals rapid inhibition of multiple tissues by old blood. Nat Commun. 2016; 7:13363. 10.1038/ncomms13363 27874859 PMC5121415

[36] Nemeth E, Tuttle MS, Powelson J, Vaughn MB, Donovan A, Ward DM, Ganz T, Kaplan J. Hepcidin regulates cellular iron efflux by binding to ferroportin and inducing its internalization. Science. 2004; 306:2090–93. 10.1126/science.1104742 15514116

[37] Du F, Qian C, Qian ZM, Wu XM, Xie H, Yung WH, Ke Y. Hepcidin directly inhibits transferrin receptor 1 expression in astrocytes via a cyclic AMP-protein kinase A pathway. Glia. 2011; 59:936–45. 10.1002/glia.21166 21438013

[38] Du F, Qian ZM, Luo Q, Yung WH, Ke Y. Hepcidin suppresses brain iron accumulation by downregulating iron transport proteins in iron-overloaded rats. Mol Neurobiol. 2015; 52:101–14. 10.1007/s12035-014-8847-x 25115800

[39] Brasse-Lagnel C, Karim Z, Letteron P, Bekri S, Bado A, Beaumont C. Intestinal DMT1 cotransporter is down-regulated by hepcidin via proteasome internalization and degradation. Gastroenterology. 2011; 140:1261–1271.e1. 10.1053/j.gastro.2010.12.037 21199652

[40] Harley CB, Futcher AB, Greider CW. Telomeres shorten during ageing of human fibroblasts. Nature. 1990; 345:458–60. 10.1038/345458a0 2342578

[41] Hastie ND, Dempster M, Dunlop MG, Thompson AM, Green DK, Allshire RC. Telomere reduction in human colorectal carcinoma and with ageing. Nature. 1990; 346:866–68. 10.1038/346866a0 2392154

[42] Prowse KR, Greider CW. Developmental and tissue-specific regulation of mouse telomerase and telomere length. Proc Natl Acad Sci USA. 1995; 92:4818–22. 10.1073/pnas.92.11.4818 7761406 PMC41798

[43] Cherif H, Tarry JL, Ozanne SE, Hales CN. Ageing and telomeres: a study into organ- and gender-specific telomere shortening. Nucleic Acids Res. 2003; 31:1576–83. 10.1093/nar/gkg208 12595567 PMC149817

[44] Hentze MW, Muckenthaler MU, Galy B, Camaschella C. Two to tango: regulation of Mammalian iron metabolism. Cell. 2010; 142:24–38. 10.1016/j.cell.2010.06.028 20603012

[45] Conboy MJ, Conboy IM, Rando TA. Heterochronic parabiosis: historical perspective and methodological considerations for studies of aging and longevity. Aging Cell. 2013; 12:525–30. 10.1111/acel.12065 23489470 PMC4072458

[46] Xiao DS, Ho KP, Qian ZM. Nitric oxide inhibition decreases bleomycin-detectable iron in spleen, bone marrow cells and heart but not in liver in exercise rats. Mol Cell Biochem. 2004; 260:31–37. 10.1023/B:MCBI.0000026048.93795.03 15228083

[47] Zhou YF, Wu XM, Zhou G, Mu MD, Zhang FL, Li FM, Qian C, Du F, Yung WH, Qian ZM, Ke Y. Cystathionine β-synthase is required for body iron homeostasis. Hepatology. 2018; 67:21–35. 10.1002/hep.29499 28859237

[48] Qian ZM, Xiao DS, Tang PL, Yao FY, Liao QK. Increased expression of transferrin receptor on membrane of erythroblasts in strenuously exercised rats. J Appl Physiol (1985). 1999; 87:523–29. 10.1152/jappl.1999.87.2.523 10444608

[49] Xiao DS, Qian ZM. Plasma nitric oxide and iron concentrations in exercised rats are negatively correlated. Mol Cell Biochem. 2000; 208:163–66. 10.1023/A:1007062626218 10939641

[50] Qian ZM, Xiao DS, Ke Y, Liao QK. Increased nitric oxide is one of the causes of changes of iron metabolism in strenuously exercised rats. Am J Physiol Regul Integr Comp Physiol. 2001; 280:R739–43. 10.1152/ajpregu.2001.280.3.R739 11171652

[51] Qian ZM, Chang YZ, Leung G, Du JR, Zhu L, Wang Q, Niu L, Xu YJ, Yang L, Ho KP, Ke Y. Expression of ferroportin1, hephaestin and ceruloplasmin in rat heart. Biochim Biophys Acta. 2007; 1772:527-532. 10.1016/j.bbadis.2007.02.006 17383861

[52] Chen Y, Qian ZM, Du J, Duan X, Chang Y, Wang Q, Wang C, Ma YM, Xu Y, Li L, Ke Y. Iron loading inhibits ferroportin1 expression in PC12 cells. Neurochem Int. 2005; 47:507–13. 10.1016/j.neuint.2005.06.004 16095759

[53] Romero-Calvo I, Ocón B, Martínez-Moya P, Suárez MD, Zarzuelo A, Martínez-Augustin O, de Medina FS. Reversible Ponceau staining as a loading control alternative to actin in Western blots. Anal Biochem. 2010; 401:318–20. 10.1016/j.ab.2010.02.036 20206115

[54] Wu XM, Qian ZM, Zhu L, Du F, Yung WH, Gong Q, Ke Y. Neuroprotective effect of ligustilide against ischaemia-reperfusion injury via up-regulation of erythropoietin and down-regulation of RTP801. Br J Pharmacol. 2011; 164:332–43. 10.1111/j.1476-5381.2011.01337.x 21410687 PMC3174414

[55] Huang XT, Qian ZM, He X, Gong Q, Wu KC, Jiang LR, Lu LN, Zhu ZJ, Zhang HY, Yung WH, Ke Y. Reducing iron in the brain: a novel pharmacologic mechanism of huperzine A in the treatment of Alzheimer’s disease. Neurobiol Aging. 2014; 35:1045–54. 10.1016/j.neurobiolaging.2013.11.004 24332448

[56] Cawthon RM. Telomere measurement by quantitative PCR. Nucleic Acids Res. 2002; 30:e47. 10.1093/nar/30.10.e47 12000852 PMC115301

[57] Gadalla SM, Wang T, Haagenson M, Spellman SR, Lee SJ, Williams KM, Wong JY, De Vivo I, Savage SA. Association between donor leukocyte telomere length and survival after unrelated allogeneic hematopoietic cell transplantation for severe aplastic anemia. JAMA. 2015; 313:594–602. 10.1001/jama.2015.7 25668263 PMC4388056

