# Supplementary material for: Tissue iron is negatively correlated with TERC or TERT mRNA expression: a heterochronic parabiosis study in mice
Source: Aging (Albany NY). 2018 Dec 16;10(12):3834–50. doi: 10.18632/aging.101676 (PMC6326661; doi:10.18632/aging.101676)
Supplement: Supplementary Figure 1 [file aging-10-101676-s001.pdf]

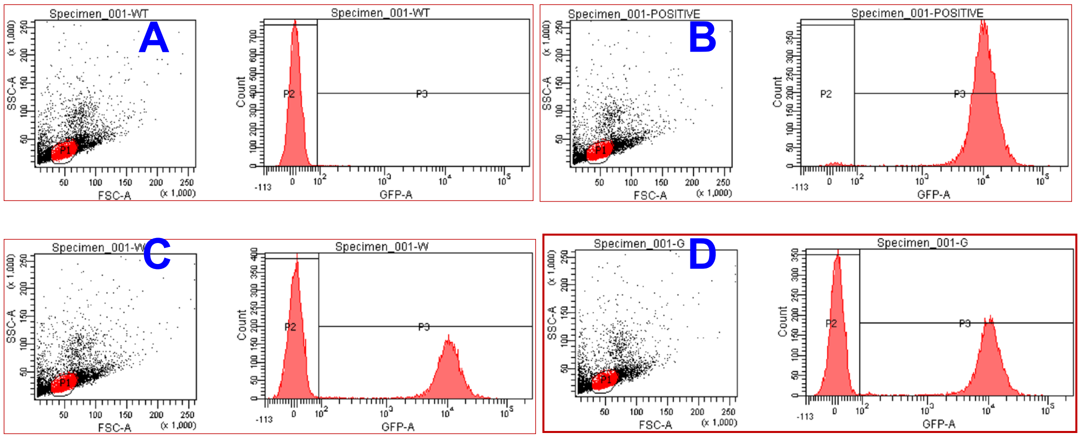

E

Group	Chimerism (%)
Wild type	42
CAG-GFP transgenic	47

F

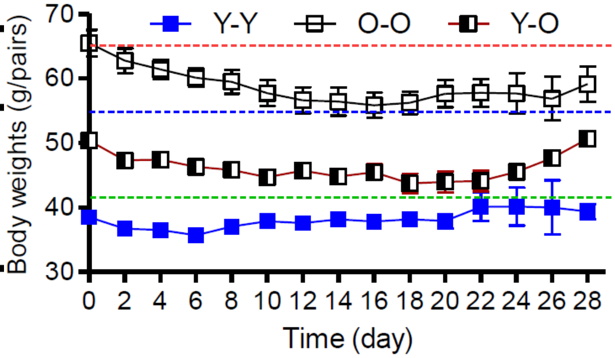

Supplementary Figure 1. Blood chimerism of a heterochronic parabiotic model. To verify the blood chimerism in parabiotic mice, GFP mice were used and the percentage of GFP$^+$ cells in the spleen was analyzed via flow cytometry in each wild-type animal connected to the GFP$^+$ parabiotic partner after 4 weeks, postoperatively. Non-connected wild type or GFP mice were seen as negative (**A**) and positive controls (**B**). Flow cytometry analysis showed that the blood chimerism of parabiotic wild type (**C**) and GFP (**D**) mice was 42% and 47% respectively (**E**), consistent with the establishment of parabiotic cross-circulation. Data shown in F are changes in the body weights of Y-O, Y-Y and O-O mice.
