# Supplementary material for: Tissue iron is negatively correlated with TERC or TERT mRNA expression: a heterochronic parabiosis study in mice
Source: Aging (Albany NY). 2018 Dec 16;10(12):3834–50. doi: 10.18632/aging.101676 (PMC6326661; doi:10.18632/aging.101676)
Supplement: Supplementary Table 1 [file aging-10-101676-s002.pdf]

Supplementary Table 1. Primer sequences.

Names	Sequences(5'-3')
β-actin	Forward AAATCGTGCGTGACATCAAAGA
	Reverse GCCATCTCCTGCTCGAAGTC
TERC	Forward TGTGGGTTCTGGTCTTTTGTTCTCCG
	Reverse GTTTTTGAGGCTCGGGAACGCG
TERT	Forward GGATTGCCACTGGCTCCG
	Reverse TGCCTGACCTCCTCTTGTGAC
Telomere	Forward GGTTTTTGAGGGTGAGGGTGAGGGTGAGGGTGAGGGT
	Reverse TCCCGACTATCCCTATCCCTATCCCTATCCCTATCCCTA
36B4	Forward CTCACTCCATCATCAATGGATACAA
	Reverse CAGCCAGTGGGAAGGTGTAGTCA
